# Stress-mediating inflammatory cytokine profiling reveals unique patterns in malaria and typhoid fever patients

**DOI:** 10.1371/journal.pone.0306585

**Published:** 2025-02-27

**Authors:** MacDonald Bin Eric, Palmer Masumbe Netongo, Severin Donald Kamdem, Christine Nzuno, Ange Maxime Tchoutang, Tchoupe Kamoua Eric Berenger, Bongkiyung Donald Buri, Ngum Leslie Ngum, Jean Paul Chedjou, Akindeh Mbu Nji, Wilfred Fon Mbacham

**Affiliations:** 1 Department of Biochemistry, University of Yaoundé 1, Yaoundé, Cameroon; 2 Molecular Diagnostics Research Group, Biotechnology Centre, University of Yaoundé 1, Yaoundé, Cameroon; 3 Laboratory for Public Health Research Biotechnologies, University of Yaoundé 1, Yaoundé, Cameroon; 4 School of Science, Navajo Technical University, Crownpoint, New Mexico, United States of America; 5 Department of Pathology, University of Utah School of Medicine, Utah, Salt Lake City, United States of America; 6 Department of Medical Laboratory Sciences, University of Buea, Buea, Cameroon; 7 Faculty of Medicine and Biomedical Sciences, University of Yaoundé 1, Yaoundé, Cameroon; 8 Institute for Medical Research and Medicinal Plants Studies, Yaoundé, Cameroon; 9 Department of Biochemistry, Faculty of Science, University of Buea, Buea, Cameroon; University of Health and Allied Sciences, GHANA

## Abstract

Malaria and typhoid fever pose significant health risks, leading to severe morbidity and mortality when inadequately treated. Understanding the role of stress-related inflammatory cytokines is crucial, as they mediate immune responses that affect pathogen clearance and recovery. This study investigated the cytokine profiles in patients with malaria and/or typhoid fever attending the Obala District Hospital in Yaoundé, Cameroon. We conducted a cross-sectional observational study measuring cortisol and inflammatory cytokines in blood samples from 55 infected patients and a control group of 15 healthy individuals using ELISA kits. We also evaluated psychological stress over the past 30 days using a 10-item Perceived Stress Scale (PSS) questionnaire to explore the link between stress and immune response. Psychological stress levels were notably higher in the typhoid fever group (18.20 ± 5.5) compared to the other groups, although these differences were statistically insignificant. Cortisol levels were significantly elevated (p < 0.001) across all patient groups compared to controls, with the typho-malaria group demonstrating a 2.5-fold increase. Notably, cytokine levels were elevated in patients with malaria and typhoid comorbidity, particularly IL-1β, IL-2, TNF-α, and IFN-γ. While IL-6 concentrations were significantly higher in malaria and typho-malaria co-infected patients, IL-10 levels were reduced in the typho-malaria group but remained elevated compared to controls. The TNF-α/IL-10 ratio was significantly higher in the co-infected group, suggesting a heightened inflammatory response. Additionally, there was a positive correlation between perceived stress scores and IL-2 (r = 0.365, p = 0.002), IFN-γ (r = 0.248, p = 0.03), and IL-6 (r = 0.412, p = 0.0001) in the typho-malaria group. Beyond IL-6, no significant correlations were observed between stress indices and the anti-inflammatory cytokines IL-4 (r = 0.204, p = 0.09) and IL-10 (r = 0.153, p = 0.20) among co-infected individuals. These results suggest that stress response may play a crucial role in shaping the inflammatory landscape during malaria and typhoid fever. Exposure to severe stressors may disrupt immune response and contribute to negative health outcomes. Understanding the immunopathogenesis of these diseases could potentially pave the way for the development of novel therapeutic strategies targeting the stress-cytokine axis.

## Introduction

Malaria is a serious disease that affects millions of people worldwide and is caused by parasites transmitted through the bite of *Plasmodium*-infected mosquitoes. According to recent data from the World Health Organization, there were an estimated 249 million malaria cases in 2022, resulting in approximately 608 000 deaths [1]. It remains a leading cause of morbidity and mortality in many parts of the world. On the other hand, the *Salmonella enterica serotypes Typhi, Paratyphi A, B*, and *C* cause potentially severe and occasionally life-threatening bacteremic illnesses collectively referred to as enteric fever. Typhoid fever outbreaks have been reported in many developing countries, particularly in areas with large populations and inadequate health infrastructures [2]. In addition, the emergence of antibiotics-resistant strains of *Salmonella* species has made controlling the disease more challenging. WHO in 2021, reported about 11–21 million cases of typhoid fever and 5 million cases of paratyphoid fever worldwide each year, causing an estimated 135 000 – 230 000 deaths.

The co-occurrence of malaria and typhoid fever has become increasingly common due to overlapping risk factors such as poor sanitation, limited access to clean water, food, healthcare, and climatic conditions facilitating vector breeding. The burden of these diseases especially in Cameroon, involves not only the physical suffering experienced by those infected but also the economic and social impacts on the affected communities, For individual patients, both malaria and typhoid fever typically cause a wide range of overlapping symptoms [3]. In rare cases, patients may develop life-threatening complications. This similarity in clinical features between the two diseases leads individuals who practice symptom-based treatment without prior diagnosis to treat one disease as the other [4].

Cytokines, as key components of the immune system, play a crucial role in the body’s response to malaria and typhoid fever. However, the immunopathogenesis of these diseases is still not well elucidated. During Plasmodium infections, pro-inflammatory cytokines such as tumor necrosis factor (TNF), interleukin-1 (IL-1), and interleukin-6 (IL-6) are released from immune cells in response to parasite antigens [5,6]. Elevated levels of these cytokines have been associated with the severity of malaria symptoms and complications, such as cerebral malaria [5]. TNF, in particular, has been shown to mediate pathological effects during severe malaria by increasing vascular permeability, activating endothelial cells, and promoting leukocyte adhesion [7]. In the case of typhoid fever, pro-inflammatory cytokines, including IFN-γ, IL-6, IL-10, TNF-α, and TNF, are also produced in large amounts [8,9]. High concentrations of these cytokines have been linked to typhoid fever complications [10]. Thus, an excessive inflammatory response mediated by cytokines like TNF, IL-1, and IL-6 likely contributes to the tissue damage and organ dysfunction observed in severe malaria and typhoid fever.

A recent study demonstrated a fundamental relationship between the immune and endocrine systems in modulating an adequate response to physiological and psychological stressors [11]. Although the exact mechanisms governing the interplay between cortisol and cytokines are still not fully elucidated, the reciprocal interaction between cortisol and inflammatory cytokines is crucial for maintaining the delicate balance between the hypothalamic–pituitary–adrenal [HPA] axis and the immune system, ensuring optimal functioning of both systems during infection. Identifying biomarkers linked to stress and immune dysregulation may enhance diagnostic and treatment strategies, ultimately improving patient care and outcomes. This study was therefore designed to investigate the effect of malaria, typhoid and comorbidity on inflammatory cytokine concentrations in patients diagnosed with typhoid fever and/or malaria.

## Research methods

### Study design

We conducted a cross-sectional observational study involving patients diagnosed with malaria and/or typhoid fever seeking medical attention at the Obala District (rural) hospital in Yaoundé, Cameroon from September, 2022 to June, 2023.

### Sample size

In order to balance between study feasibility and statistical power, minimise risk of dropouts, a convenient sample size of 70 participants (55 malaria and/or typhoid fever confirmed cases and 15 healthy controls) were recruited.

### Patient selection and clustering

**Fig 1:**
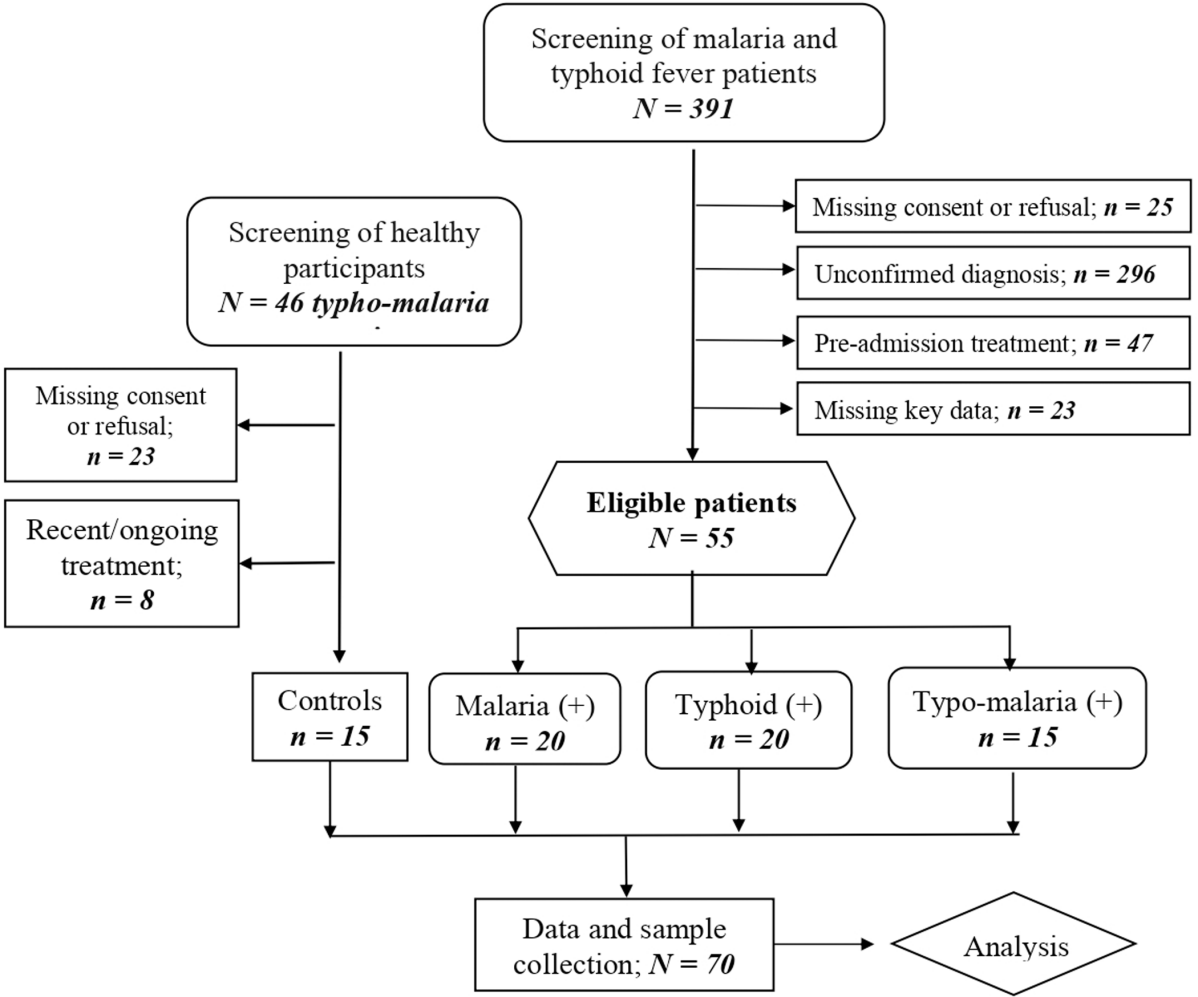
The study flowchart indicating the strategy of selection and clustering of participants.

We included voluntary participants of age 10 years and above, who provided written informed consent and assent. The patients were selected based on their infectious status of either malaria or typhoid fever (Fig 1). A stratified random sampling technique was used where the population was divided into four groups [strata]: malaria (+), typhoid (+), typho-malaria (+) and Healthy control. Individuals with similar characteristics suffering from different diseases were paired to help control for confounding variables. We excluded pregnant women, patients with severe complications of malaria or typhoid fever, HIV-positive patients, other known comorbidities such as diabetes, hepatitis. These conditions can influence the stress/immune responses in patients with typhoid fever and/or malaria by altering the disease severity.

### Data collection

Participants’ data were collected by trained clinical personnel using a standardized case report form (CRF). Data collected included demographic information, patient history, symptoms & signs (type and severity), co-morbidities, initial diagnosis, drugs and test prescribed, laboratory results and care pathway (hospitalization or outpatient care).

### Stress event measurements

We collected information about stressful life events which had occurred in the previous month using a 10-item Perceived Stress Scale [PSS) [12] on a 5-point scale (0 = never, 1 = almost never, 2 = once in a while, 3 = often, 4 = very often). A perceived stress score ranging from 0–13 was considered “low stress”, 14–26 as “moderate stress” and 27–40 as high perceived stress.

### Sample collection and laboratory analysis

Patients with signs and symptoms presumptive of malaria and/or typhoid fever were clinically screened and suspected participants were prescribed both malaria and typhoid fever tests. After the patient had been clinically diagnosed for malaria and/or typhoid fever, about 15 ml of blood sample were collected. Blood samples were then distributed in EDTA, dry and blood culture tubes. All laboratory analyses were performed by competent laboratory personnel.

### Diagnosis and confirmation of *Plasmodium* infection

Malaria diagnosis was done using standard diagnostic methods as described elsewhere [4]. Malaria Rapid Diagnostic Test kits (CareStart^TM^, ACESSBIO) were used to detect the presence of *Plasmodium* antigens in the patient’s blood in accordance with the manufacturer’s instructions. Thick and thin blood films were also prepared and stained with Giemsa for a confirmatory diagnosis of malaria and parasite count using microscopy. Two experienced laboratory personnel independently examined the slides for the presence of *Plasmodium* parasites.

### Diagnosis and confirmation of typhoid fever

#### Rapid diagnosis of typhoid fever.

Package inserted protocol instructions were followed. The laboratory screening of typhoid fever was done using the OnSite Typhoid IgG/IgM Combo rapid test (CTK Biotech Inc, USA), a lateral flow chromatographic immunoassay that detects and distinguishes IgG and IgM antibodies to *Salmonella typhi* and *S. paratyphi* in human blood. Results were interpreted as described elsewhere [13].

#### Widal agglutination test.

Widal test was performed on acute serum using Sanofi qualitative agglutination test kits (Bio-Rad) containing coated somatic (O) and flagella (H) antigens of *S. typhi* and *S. Paratyphi A, B* and *C*. Analysis was initially carried out as described previously [4]. All positive results obtained through a slide test were confirmed and quantify by the tube agglutination method. A positive Widal test was considered as one that gave a reaction titre greater or equal to 1/200 for *Salmonella* somatic and flagella antibodies, after the prescribed incubation time [14]. However, the last test showing signs of agglutination was taken as the titre for that patient [15].

#### Culture and identification of *Salmonella spp.*

About 4 ml of venous blood sample collected from each participant was immediately inoculated into bottle containing 46 ml triptic soya broth medium (Himedia, India) and incubated for 7 days. The cultured bottle which showed growth were further sub cultured on MacConky agar (Deben diagnostic Ltd) and blood agar media (Biomark, India laboratories) after 48 h. Negative broth culture were incubated for seven days and sub cultured before reported negative. Specific antisera were used to determine *Salmonella spp.*

### Estimation of inflammatory cytokines levels

Blood samples were transferred to the Molecular Diagnostic Research (MDR) Laboratory (Yaoundé, Cameroon) and Laboratory for Public Health Research Biotechnologies (LAPHER BIOTECH) at the Biotechnology Centre (University of Yaoundé I, Cameroon) within 2 h, in cooled bags, then centrifuged for 10 min at 3500 rpm at 4 °C to allow plasma and serum collection. Seven inflammatory cytokines were assayed in duplicate using ELISA: interleukin (IL)-1β (Cat #: EKHU-0083, Melsin, China), IL-2 (Cat #: EKHU-0144, Melsin, China), IL-4 (Cat #: EKHU-0014, Melsin, China), IL-6 (Cat #: EKHU-0140, Melsin, China), IL-10 (Cat #: EKHU-0155, Melsin, China), tumor necrotic factor (TNF)-α (Cat #: EKHU-0110, Melsin, China), and interferon (IFN) -γ (Cat #: EKHU-1695, Melsin, China).

Following the manufacture’s guide, the standard cytokine was diluted in assay buffer to obtain varying concentrations in ng/L depending on the biomarker. The dilutions were then pipetted into corresponding wells followed by addition of test serum to each well containing a standard dilution, along with sample diluent, and a sample blank well was prepared containing only chromogen solution A and B, stop solution, and no serum.

The plate was incubated at 37°C for 30 minutes, with HRP-conjugate reagent added to all wells except the blank well. After incubation, the plate was washed four times with wash buffer to remove any unbound reagents. Chromogen solution A and B were then added to each well, mixed well, and incubated at 37°C for an additional 10 minutes. The stop solution was finally added to each well, and the optical density (OD) was measured using a microtiter plate reader at a wavelength of 450 nm within 15 minutes. The OD values were set to zero for the blank well. A standard curve of OD against cytokine concentration was plotted using the prepared standards, and this curve was used to determine the concentration of the test samples.

### Assessment of stress response

The level of cortisol was estimated using commercially available enzyme-linked immunosorbent assay (ELISA) kit (Cat #: ARG8139, Arigo BioLaboratories, Taiwan). All blood parameters were tested following the manufacturer’s instructions.

Samples were prepared by mixing 5 µL of plasma with 5 µL of dissociation reagent, followed by the addition of 490 µL of assay buffer. Standards and diluted samples were added to a microplate, along with assay buffer in the non-specific binding well. Cortisol conjugate and cortisol antibody were then added to the other wells, with the plate shaken for one hour at room temperature. After washing the wells four times with wash buffer, TMB substrate solution was added and incubated for 30 minutes. The reaction was halted with stop solution, and the optical density measured at 450 nm using a microplate reader to quantify cortisol levels.

### Ethical considerations

This study was conducted in compliance with the national and international ethical standards for research involving human participants. The study protocol was reviewed and approved by ethical review committee and regulatory authorities: the Centre Regional Ethics Committee for Human Health Research (Ref. #: 0226/CRERSHC/2022) and the Centre Regional Delegation of Public Health under the ministry of Public Health in Cameroon (Ref. #: 1392/AAR/MINSANTE/SG/DRSPC). Prior to data collection, informed consent was obtained from all participants. The consent process included a detailed explanation of the study’s purpose, procedures, risks, and benefits, as well as participants’ right to withdraw at any time without penalty. Participants were assured of their anonymity and the confidentiality of their data. All identifying information was coded and stored separately from the research data to further protect participant privacy any potential risks or adverse effects were greatly minimized.

### Data analysis

Statistical analysis was performed using SPSS (V.26) and GraphPad (Prism Software 9.0.0). The Kruskal-Wallis H test was utilized to compare the cytokine concentrations across the groups. Also, Spearman rank correlation coefficients were calculated to assess the relationship between cytokine concentrations and cortisol levels among patients, as well as to examine how these levels correlate with hematological parameters. The differences were considered statistically significant if p < 0.05.

## Results

### Assessment of general demographic characteristics of the study population

The general demographic characteristics of patients (n = 55) and healthy controls (n = 15) are presented in Table 1. Among eligible patients, the female-to-male ratio was 1.04, with a mean age of 25.06 years; whereas among controls, the female-to-male ratio was 1.5, with a mean age of 30.60 years. Aside from fever, the most frequently occurring symptoms in the study population were headache (54.55%) and dizziness (49.09%). The mean time from onset of symptoms to admission was highest in typhoid fever mono-infections, 4.95 (±2.54) days. Upon admission, the mean perceived stress of the co-infected group was approximately one and a half times greater than that of the control group and nearly similar to that of the typhoid fever group, with no significant difference between groups.

**Table 1:** Baseline characteristics of the study population.

Characteristics (N = 70)		Healthy control *(n = 15)*	Malaria mono *(n = 20)*	Typhoid mono *(n = 20)*	Typho-malaria *(N = 15)*	*p*-value
Mean age (±SD)/years		30.60 (±12.19)	22.35 (±14.54)	27.50 (±9.50)	25.33 (±10.65)	0.228
Gender	Male, **n (%)**	6 (40.00)	12 (60.00)	7 (35.00)	8 (53.33)	0.211
	Female, **n (%)**	9 (60.00)	8 (40.00)	13 (65.00)	7 (46.67)	
Core temperature (SD)/^o^C		36.7 (±0.38)	**38.44 (±0.67)** *	**38.55 (±1.09)** *	**39.07 (±1.18)** *	**<0.001**
Clinical presentation on admission						
Headache, n (%)		/	11 (55.00)	8 (40.00)	11 (73.33)	0.224
Anorexia, n (%)		/	9 (45.00)	5 (25.00)	7 (46.67)	0.157
Sweating, n (%)		/	2 (10.00)	3 (15.00)	4 (26.67)	0.212
Dizziness, n (%)		/	8 (40.00)	9 (45.00)	10 (66.67)	0.084
Body pain, n (%)		/	7 (35.00)	4 (20.00)	**10 (66.67)** *	**0.024**
Vomiting, n (%)		/	4 (20.00)	2 (10.00)	3 (20.00)	0.131
Diarrhea, n (%)		/	4 (20.00)	3 (15.00)	9 (60.00)*	**0.029**
Abdominal pain, n (%)		/	2 (10.00)	6 (30.00)	8 (53.33)	0.214
Anaemia, n (%)		/	**6 (30.00)** *	2 (3.64)	**6 (10.91)** *	**<0.001**
Mean onset of symptoms (SD)/days		–	2.10 (±1.55)	4.95 (±2.54)	4.00 (±1.73)	0.147
Average weight (SD)/kg		64.64 (±10.59)	50.97 (±19.19)	**64.34 (±10.57)** *	57.46 (±13.33)	**0.042**
Mean systolic BP (SD)/mmHg		115.53 (±3.85)	**108.80 (±6.46)** *	**109.60 (±8.04)** *	114.07 (±6.97)	**<0.01**
Mean diastolic BP/mmHg		65.73 (±2.96)	**59.85 (±5.95)** *	64.35 (±5.81)	62.80 (±8.11)	**0.027**
Median parasite density (range)/µl		/	1850.00 (105–103025)	/	**12850** **(1035–122045)** *	**<0.001**

Data represented as count (percentage, %), mean (standard deviation) and range (min. - max.) where the asterisk

(*) corresponds to statistical signiﬁcance at p < 0.05.

### Laboratory ﬁndings

#### Significant hematological alterations among patients compared to controls.

The results of hematological parameters are shown in Table 2. Malaria patients in this study showed a mild state lymphocytopenia with less than 1000 lymphocytes per microliter of blood. Anemia, thrombocytopenia and monocytosis were equally recorded in the co-infected groups with significant differences compared to the control group.

**Table 2:** Changes in hematological parameters associated with malaria, typhoid and typho-malaria comorbidity.

Blood parameter	Control n = 15	Malaria mono n = 20	Typhoid mono n = 20	Typho-malaria co-infection, n = 15	p-value
*Leukocyte (SD) ×10* ^*9*^ */L*	4.51 (±0.51)	**5.08 (±0.78)** *	4.17 (±1.03)	4.46 (±1.15)	**0.021**
*Hematocrit, (SD) %*	43.78 (±2.70)	40.8 (±2.75)	41.9 (±2.36)	**38.47 (±3.54)** *	**<0.001**
*Neutrophils (SD) ×10* ^*9*^ */L*	1.97 (±0.24)	2.13 (±0.37)	1.95 (±0.29)	1.84 (±0.37)	0.079
*Lymphocytes (SD) ×10* ^*9*^ */L*	1.52 (±0.50)	**0.95 (±0.27)** *	1.19 (±0.35)	**0.98 (±0.21)** *	**<0.001**
*Monocytes (SD) ×10* ^*9*^ */L*	0.63 (±0.33)	0.82 (0.48)	0.99 (±0.48)	**1.15 (±0.37)** *	**0.009**
*Eosinophils (SD) ×10* ^*9*^ */L*	0.20 (±0.12)	**0.077 (±0.04)** *	0.11 (±0.06)	**0.09 (0.06)** *	**<0.001**
*Platelets (SD) ×10* ^*9*^ */L*	158.33 (±28.74)	135.15 (±19.48)	132.6 (±25.01)	**113.4 (±14.2)** *	**<0.001**
*Hemoglobin (SD), g/dL*	14.22 (±1.68)	12.11 (±1.61)	12.46 (±1.50)	**10.81 (±1.34)** *	**<0.001**

Data represented as mean (standard deviation) where the asterisk

(*) corresponds to statistical signiﬁcance.

#### Stress response in the study groups.

**Fig 2:**
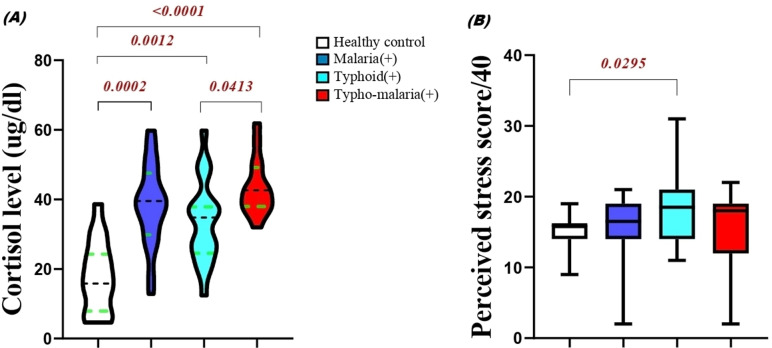
Comparison of cortisol levels (A) and perceived stress score (B) in the study groups. Data represented as concentration of cortisol in µg/dl and psychological stress score on a scale of 40 measured on admission prior to treatment.

The stress response was evaluated by estimating the cortisol within the study population and the result are shown in Fig 2A below. The overall mean baseline cortisol levels were significantly higher in all patient groups especially in typho-malaria group, 43.27 (±8.12) compared to the malaria group, 38.39 (±11.80), typhoid fever group, 33.70 (±11.83) control, 17.01 (±11.30). The cortisol level of the typhoid fever group was slightly and significantly different from that of the co-infected group (*p = 0.0413*).

Psychological stress results (Fig 2B) showed that patients with typhoid fever reported a significantly higher perceived stress levels (18.20 ± 5.5) compared to the control group (15.0 ± 2.43; p = 0.0295), patients with malaria (15.80 ± 4.39), and comorbidity (15.40 ± 5.26). Thematic analysis of the qualitative data revealed that patients with typhoid fever experienced more anxiety and fear related to the possibility of complications and treatment failure.

#### Inflammatory cytokine concentrations in patients compared to healthy controls.

**Fig 3:**
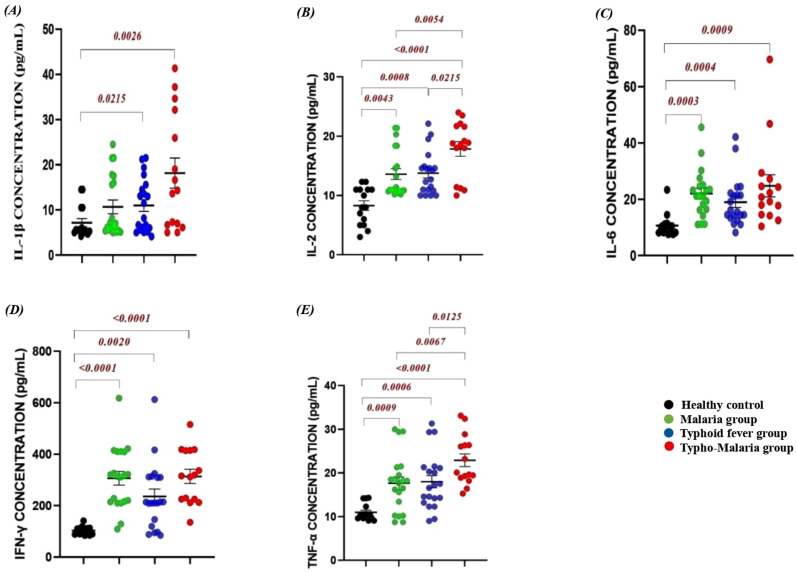
Comparison of pro-inflammatory cytokine levels in the study groups: (A) IL-1β; (B) IL-2; (C) IL-6; (D) IFN-γ and (E) TNF-α among the different groups. Data represented as concentration in pg/ml measured on admission prior to treatment.

On admission, the concentrations of IL-1β (p = 0.0026), IL-2 (p < 0.0001), IL-6 (p = 0.0009), TNF-α (p > 0.0001), and IFN-γ (p > 0.0001) were signiﬁcantly upregulated in patients with malaria and typhoid comorbidity, comparing to healthy controls (Fig 3). Aside from the significant difference in IL-2 level recorded in all patient groups, there was significant difference between typhoid group and typho-malaria group (p = 0.0215) (Fig 3B). TNF-α levels were significantly different between the mono and coinfection groups (Fig 3E).

**Fig 4:**
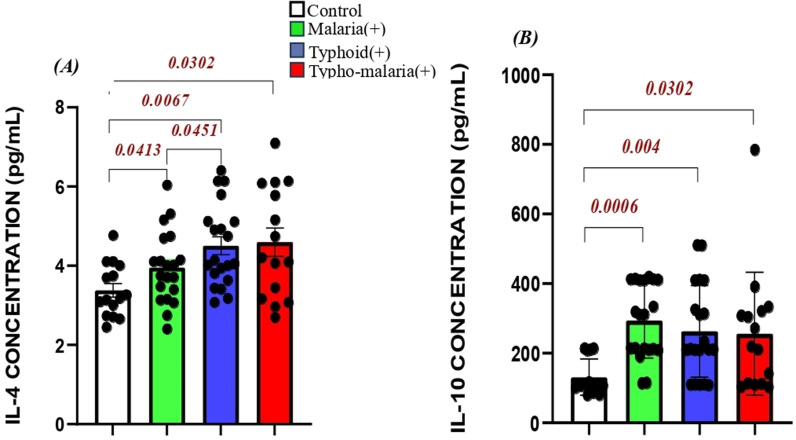
Comparison of anti-inflammatory cytokine levels in the study groups: (A) IL-4 and (B) IL-10: malaria^+^ (n = 20), typhoid^+^ (20) typo-malaria^+^ (n = 15) and Control (n = 15). Data represented as concentration in pg/ml measured on admission prior to treatment.

Anti-inflammatory cytokine levels on admission varied significantly across the groups (Fig 4). Generally, IL-4 concentrations were significantly higher in the typhoid mono and typho-malaria groups with moderate differences in other groups compared to the control (Fig 4A). On the other hand, IL-10 levels were slightly reduced in the typho-malaria group (255.3 ± 177.0) compared to the mono- malaria (293.1 ± 106.8) and typhoid fever (2262.6 ± 131.9) groups, but significantly higher when compared with control (130.9 ± 51.56). In spite the insignificant variation among patient groups, the malaria group showed a very strong significant rise in IL-10 (p = 0.0006) compared to the heathy control. The typhoid group equally recorded a significant rise in IL-10 compared to the control (p = 0.004) (Fig 4B).

#### Pro-inflammatory versus anti-inflammatory cytokine ratios compared among groups.

**Fig 5:**
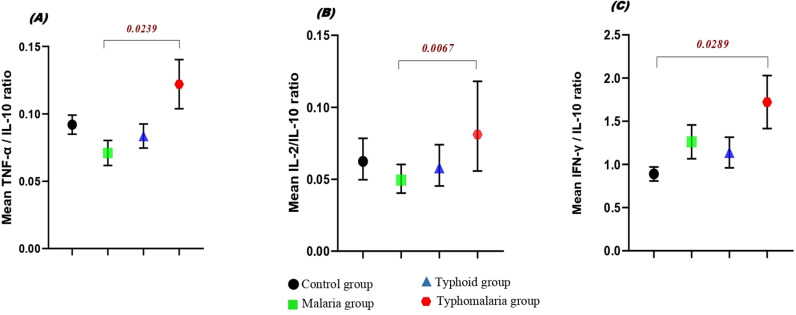
Comparison of the pro/anti-inflammatory cytokine ratios in the study groups: (A) TNF-α/IL-10; (B) IL-2/IL-10; (C) IFN-γ/IL-10.

Disbalances in pro- and anti-inflammatory cytokines were observed in the study population. Therefore, we chose pro-inflammatory cytokines (IL-2, TNF-α and IFN-γ) which act as key actors in the regulation of infections with statistical significance (*p < 0.001*) and divided the expression by IL-10 which was the key anti-inflammatory cytokine identified in the study group (*p < 0.05*) to calculate a pro- and anti-inflammatory cytokine expression ratio (PAER). Results of these ratios where equally compared between groups as shown in Fig 5. Among the patient groups, the mean IL-2/IL-10 ratio was significantly higher in typho-malaria group compared to the malaria group (p = 0.0067).

## Discussion

This study investigated the relationship between stress response and inflammatory cytokine concentrations in patients diagnosed with malaria and/or typhoid fever. This absence of demographic variability in this study minimizes bias, allowing us to attribute the observed differences to the pathogens rather than extraneous factors, which is crucial for understanding the interplay between stress response and inflammatory profiles.

Malaria patients in this study showed a mild state lymphocytopenia with less than 1000 lymphocytes per microliter of blood. Anemia, thrombocytopenia and monocytosis were equally recorded in the co-infected groups with significant difference from the control group. These observations have been reported to be significantly associated with malaria and estimated to be specific for the diagnosis of malaria [16–19]. The cause of thrombocytopenia in malaria and typhoid fever is poorly understood but, platelet indices could be useful predictors of disease severity [20,21]. Correlation analysis revealed that duration of pre-admission symptoms in typho-malaria showed strong positive correlation with diastolic blood pressure (*r = 0.421, p = 0.001*) and perceived stress index (*r = 0.369, p = 0.006*). This implies that prolonged exposure to the pathogens may lead to increased cardiovascular and psychological stress, which could alter severity and outcomes. This highlights the importance of considering the psychological toll and duration of symptoms when evaluating patients with malaria and/or typhoid fever.

Additionally, in the typho-malaria group, perceived stress index gave strong positive correlation with parasite density (*r = 0.613, p < 0.001*] and eosinophil count (r = 0.303, p = 0.01) while core temperature and eosinophil count were negatively correlated (r = −0.343, p = 0.004). This implies that stress may play a role in modulating the immune response to infection during co-infections, potentially allowing for greater parasite growth. Thus, the decreased eosinophil counts recorded in patients with malaria and typho-malaria is likely to be associated with an impaired immune response, reduced inflammatory response, and increased risk of severe disease during *Plasmodium* infection with less effect during typhoid fever.

An approximately three-fold increase in parasitemia in typho-malaria group compared to those patients with malaria only (geometric mean parasitaemia of 34711.2 versus 12744.1 per µl of blood, p value of < 0.001) was observed. A similar significantly high parasitemia was reported previously by Netongo *et al*., in 2022 [4]. This implies that co-infection enhances replication of malaria parasites, impairs immune response and favors transmission. Correlation analysis revealed a significant negative correlation between parasite density and hemoglobin count in both the malaria mono (r = −0.356, p = 0.005) and coinfected groups (r = −0.457, p < 0.001). This is consistent with the known effects of *Plasmodium* parasites on red blood cell production and destruction. It could be suggested here that the *Salmonella* organism contributes to parasite-mediated hemolysis and disruption of erythropoiesis, consistent with the known immunosuppressive effects of malaria on the bone marrow [22]. A similar result was recently reported in malaria patients by Antwi-Bafour *et al*., 2023 [23].

In this study, an overall mean cortisol level in the typho-malaria group was over 2.5 times greater than that of the control (43.27 µg/dl versus 17.01 µg/dl). This is contrary to the report of Ibrahim EA *et al*., in 2011 [24] who recorded no significant difference in mean cortisol levels in patients with malaria in comparison with the control group. Thus, stress response is activated in patients with malaria and typhoid fever and in an attempt to cope with this threat, a physiological response is triggered. Also, elevated cortisol levels may indicate dysregulation of the hypothalamus/pituitary/adrenal axis (HPA) axis, which is responsible for regulating the body’s response to stress. Elevated cortisol levels have previously been attributed to increased risk of complications in recent studies, such as hyperglycemia [25], hypertension [26], and impaired immune function [27].

Correlation analysis revealed that rise in cortisol level had a positive correlation with core temperature in all patient group with the strongest correlation in the typho-malaria group (r = 0.492, p < 0.0001). This can thus be useful in predicting severity during malaria and typhoid fever comorbidity. A current investigation confirmed that temperature interfered with the cortisol secretion; suggesting stimulation of this hormone in malaria patients [28]. On the other hand, cortisol levels showed a strong negatively correlation with hemoglobin (r = −0.352, p = 0.003) and platelet counts (r = −0.373, p = 0.02) in the typho-malaria group, with an insignificant positive correlation with leukocyte (r = 0.144, p = 0.23). This finding implies that cortisol levels may be contributing to the hemolytic anemia and thrombocytopenia commonly seen in patients with malaria and typhoid fever.

It has been documented that the blood-stage cycle of the malaria parasite is characterized by an upregulation of inflammatory cytokines like IL-6, IFN-γ, and TNF-α, which play a pivotal role in controlling the growth of the parasite and its elimination [29]. The protective immune responses to *Salmonella* infection are complex and involve both humoral and cellular immune responses [30,31], even though the role of humoral immunity in protection remains undefined [10]. Due to the ability of *Salmonella* to persist intracellularly, cell-mediated immunity is critical to clearance of infection. In this study, the concentrations of pro and anti-inflammatory cytokines varied considerably from one group to another. IL-1β levels in the malaria mono and typhoid mono groups were significantly compared to the typho-malaria group (p < 0.05). This suggests that co-infection triggered immune suppression which is consistent with the idea that co-infection can lead to immune exhaustion, where the immune system is unable to effectively respond to one or both of the pathogens. A role of IL-1β in malaria severity has been reported [32] while in the case typhoid fever, the findings are inconsistent. Lyke *et al*., in 2004 [33] reported no significant change in IL-1β attributing this to the downregulation by IL-10 levels. This could also explain why the IL-1β level in this present study was highest in typho-malaria group with a lower IL-10 level. On the contrary, the level of IL-1β was significantly lower in the malaria group with a higher level of IL-10.

It has been established that during early *Salmonella* infection, inflammatory monocytes produce anti-microbial factors such as TNF-α and IL-1β [34]. In the study, we observed a significant rise in TNF-α levels in the both the typhoid mono-and co-infected groups compared to the control. Unlike in the report of Lyke *et al*., in 2004 [33] who reported an insignificant difference in TNF-α level in malaria patients, we obtained a rather very high significant difference in this group compared to the control. TNF-α elevation has been previously associated with anemia and high-density *P. falciparum* infection [35], whereas reduced IL-10 demonstrated in African children with severe malaria-induced anemia. In our study, the mean concentration of IL-10 was signiﬁcantly higher in patients with malaria only, 293.12 (±106.80), compared to those with comorbidity, 255.30 (±176.95) and healthy controls 130.93 (±51.56). In the typhoid fever group, IL-2 was equally strongly and positively correlated to IL-6 (r = −0.464, p = 0.01). In the malaria and typhoid fever co-morbidity group, a positive correlation was recoded between IL-2/TNFα (r = 0.515, p = 0.03), IL-2/IL-6 (r = 0.536, p = 0.02) and IL-6/TNF-α (r = 0.664, p = 0.007).

Correlation between these cytokines suggests a complex interaction between immune cells in the malaria and typhoid fever co-morbidity which might contribute to disease severity as seen in the significant increase in TNF-α levels and the decrease in IL-10 levels in patients with malaria and typhoid comorbidity compared to those with malaria only.

In line with a previous report [36], we recorded a significant rise in IFN-γ levels in all patient groups with more significant increase in malaria and co-infected patients. The level of IFN-γ in typhoid fever patient was also significantly high and confirming the report by Sheikh *et al*., in 2011 [37]. This confirms the fact that IFN-γ responses against *Salmonella* antigens are elevated in both acute and convalescent stages of human infection compared with healthy controls. Correlation analysis in this study revealed that IL-2 equally gave a strong negative correlation with IFN-γ in the typho-malaria group (r = −0.577, p < 0.008). The strong negative correlation between IL-2 and IFN-γ suggests that these two cytokines have opposing effects in the immune response to typho-malaria.

A previous work revealed that, after controlling for demographic factors, a rise in IL-6 was related to increased cortisol in other conditions [11]. A. Wolkow *et al*., in 2015 [11] observed a less pronounced association between TNF-a, IL-10, IL-4, and cortisol. This suggests that the relationship between stress and anti-inflammatory cytokines may be more complex and influenced by various factors. Further investigation is needed to elucidate the underlying mechanisms by which stress modulates the cytokine profile specifically in co-infections. Unlike in the mono infected patients, cortisol level in the typho-malaria group showed a negative correlation with IL-6 levels (r = −0.411, p = 0.03), and TNF-α levels (r = −0.413, p = 0.01) suggesting a possible dampening effect of cortisol on inflammation in the co-infected group possibly due to the overwhelming immune response to the dual infection. Cortisol can sometimes have immunomodulatory effects, influencing the immune system in both pro- and anti-inflammatory [38] ways depending on the context.

Previous reports suggest that the balance between pro- and anti-inflammatory cytokines determines parasite load and disease outcome [39–41]. In contrast, other evidences suggest that disease outcome depends on cytokine overproduction and not on the balance between them, since high levels of anti-inflammatory as well as pro-inflammatory cytokines may be associated with disease severity and mortality [42]. Our findings revealed a clear distinction in the immune response between the different clusters. The co-infected group displayed a significantly higher TNF-α/IL-10 ratio, indicative of a more robust and potentially detrimental inflammatory response. Interestingly, the co-infection group showed a higher IL-2/IL-10 and IFN-γ/IL-10 ratios, indicating a more pronounced effort to activate T-cells and macrophages and an overreaction might potentially provoke a collateral damage. These findings collectively suggest that co-infection with malaria and typhoid fever leads to a more severe and dysregulated immune response compared to single infections.

## Conclusion

Our study highlights the complex interplay between stress and the immune response in patients with malaria, typhoid fever, and co-infections. The distinct cytokine profiles observed between mono-infected and co-infected patients underscore the intricacy of the immune response in these conditions. Severe stressors can disrupt immune function, leading to adverse health outcomes. Our findings emphasize the need to understand how infectious diseases, stress, and immune responses interact to develop effective treatment strategies. Future research should investigate the mechanisms through which stress affects cytokine expression and explore the therapeutic potential of stress management alongside conventional treatments for malaria and typhoid fever.
